# 

**Published:** 1869-01

**Authors:** 


### INDEX OF HALL'S JOURNAL OF HEALTH.

### VOL. XVI., 1869.

| | PAGE. |
|---|---|
| Ash Sifters, | 39 |
| Adhesive, Best, | 160 |
| Air, | 22, 271 |
| Breaking Down, | 6 |
| Bad Air, | 22 |
| Boots, Tight, | 33 |
| Breathing, | 34 |
| Bronchitis, | 126 |
| Baldwin the Great, | 171 |
| Baldness, | 179 |
| Beautiful Saying, | 186 |
| Bathing, | 200 |
| Bungling, | 200 |
| Bread, Good | 222 |
| Boat-racing, | 247 |
| Biliousness, | 269 |
| Boy's Success, | 278 |
| Be Useful, | 303 |
| Croup, | 4 |
| Cough, | 4 |
| Cook, A Good, | 7 |
| Chapped Hands, | 12 |
| Cheese and Nuts, | 13 |
| Christian Associations, | 14 |
| Child Murder, | 55 |
| Common Cold, | 59 |
| Common Sickness, | 61 |
| Cheap Living, | 62 |
| Changing Clothing, | 109 |
| Consumption, | 125, 182, 264 |
| Cell Development, | 137 |
| Costiveness, | 175 |
| Colds Prevented, | 176 |
| Causes of Disease, | 184 |
| Comfort, | 191 |
| Coffee, | 213, 273 |
| Cornaro, | 235 |
| Clergymen, | 261 |
| Chloroform, | 275 |
| Daughters, Domestic, | 6 |
| Dangerous Medication, | 17 |
| Disease, its Essence, | 25 |
| Dyspeptic, | 177 |
| Disinfectant, | 195 |
| Diarrhœa, | 196 |
| Dieting Wisely, | 235 |
| Drunkenness, | 242 |
| Dixon's Grate, | 254 |
| Daughters, | 291 |
| Dangerous Receipts, | 311 |
| Eight Hours a Day, | 149 |
| Eating by Rule, | 192 |
| Eye-sight, | 194 |
| Erect Position, | 197 |
| Emergencies, | 205 |
| Early Rising, | 211 |
| Exercise, | 272 |
| Endurance, | 289 |
| Fingers Cracked, | 13 |
| Face and Hands, | 32 |
| Feet, Cold, | 36 |
| Fire on the Hearth, | 42 |
| Fun and Physic, | 165 |
| Fair Dealing, | 171 |
| Food Items, | 185 |
| Forethought, | 205 |
| Fever and Ague, | 255 |
| Food Cure, | 296 |
| First Wrong Step, | 301 |
| Going Abroad, | 62 |
| Glue, | 160 |
| Games, | 187, 248 |
| Greatest and Best, | 201 |
| Grates, The Best, | 255 |
| Houses, Unhealthful, | 40 |
| Heidelberg, | 63 |
| Hydrophobia, | 73 |
| Hair Preservers, | 179 |
| Health and Disease, | 231 |
| Hallucination, | 275 |
| Hydrophobia, | 276 |
| Hard to Take, | 290 |
| Instinct, | 233 |
| Intermittents, | 255 |
| Ill-temper, | 274 |
| Kerosene Fatalities, | 43 |
| Kindness Rewarded, | 304 |
| Living Unwisely, | 9 |
| Life Sacrificed, | 19 |

| | PAGE. |
|---|---|
| Liebig's Soup, | 20 |
| Loose Bowels, | 175 |
| Late Eating, | 268 |
| Lungs, | 271 |
| Mad Dogs, | 73 |
| Mother's Influence, | 77 |
| May Moving, | 87 |
| Magnetism, | 103 |
| Marrying Kindred, | 108 |
| Man Described, | 108 |
| Melons, | 210 |
| Nuts and Cheese, | 13 |
| Night Air, | 224 |
| Out-door Activities, | 265 |
| "Protestantism," | 51 |
| Pulpit and Pew, | 79 |
| Preaching Well, | 97 |
| Philosopher, A, | 106 |
| Paddling Canoes, | 111 |
| Perspiration, | 176 |
| Perfumes, | 212 |
| Pipe, The Last, | 251 |
| Responsibilities, | 76 |
| Resignation, | 105 |
| Robbie's Definition, | 109 |
| Reproving Wisely, | 113 |
| Recreations, | 119 |
| Racing, | 247 |
| Restless Nights, | 277 |
| Rail Travel, | 309 |
| Rejuvenation, | 310 |
| Stomach Cough, | 3 |
| Servant's House, | 5, 251 |
| Soup, | 20 |
| Selfishness, | 29 |
| Slippers, Best, | 37 |
| Sleeping Habits, | 41 |

| | PA[illegible] |
|---|---|
| St. Albans, | [illegible] |
| Spring Diseases, | 90 |
| Stewart's Philanthropy, | 82 |
| Starved to Death, | 133 |
| Suicide, | 180 |
| Sabbath Day, | 188 |
| Stupidities, | 190 |
| Sitting Erectly, | 199 |
| Stammering, | 217 |
| Summerings, | 219 |
| Sewerage, | 226 |
| Story of a Man, | 245 |
| Smoking, | 249 |
| Stomach, | 268 |
| Sleeplessness, | 277 |
| Spectacles, | 279 |
| Sons, | 291 |
| Throat Tickling, | 1 |
| Throat Disease, | 11 |
| Table Manners, | 114 |
| Throat Ail, | 127 |
| Tomatoes, | 210 |
| Trade Strikes, | 244 |
| Travelling for Health, | 269 |
| Tea and Coffee, | 273 |
| Tom-boys, | 305 |
| Travelling, | 309 |
| Voice, | 98 |
| Vinegar, Good, | 187, 278 |
| Ventilation, | 253 |
| Vegetarianism, | 274 |
| Wakefulness | 60 |
| Walking Erectly, | 110, 198 |
| Working Men, | 149 |
| Woman's Rights, | 158 |
| Warming Houses, | 253 |
| Wet Blankets, | 306 |
| Wreck of All, | 308 |

